# Commentary: Efficacy of probiotics/synbiotics supplementation in patients with chronic kidney disease: a systematic review and meta-analysis of randomized controlled trials

**DOI:** 10.3389/fnut.2024.1510662

**Published:** 2024-12-20

**Authors:** Jakub Ruszkowski, Alicja Dębska-Ślizień

**Affiliations:** Department of Nephrology, Transplantology, and Internal Medicine, Faculty of Medicine, Medical University of Gdańsk, Gdańsk, Poland

**Keywords:** chronic kidney disease, probiotics, gut microbiota, meta-analysis, statistical issues, minimal clinically important difference, scientific method

## 1 Introduction

The article “Efficacy of probiotics/synbiotics supplementation in patients with chronic kidney disease: a systematic review and meta-analysis of randomized controlled trials” written by Chang Liu et al. (1) aimed to investigate the effects of probiotics or synbiotics supplementation on kidney function, lipid metabolism, inflammation, uremic toxin levels and electrolyte levels in patients with chronic kidney disease (CKD).

While the authors have conducted a review and meta-analysis on several important outcomes and provided mind-provoking subgroup analyses, there are several critical issues that, in our opinion, compromise the validity and clinical applicability of their findings.

## 2 Considerations regarding aggregation of interventions

The study's approach of combining probiotics and synbiotics into a single analysis group raises important methodological questions. Probiotics, which consist of live microorganisms that, when administered in adequate amounts, confer a health benefit on the host, and synbiotics, which are a combination of probiotics and prebiotics, have fundamentally different compositions and mechanisms of action. Generalizing the effects across such a diverse group is scientifically questionable and may obscure important differences in efficacy and safety.

The recent recommendations outlined in the 2023 consensus statement by McFarland et al. emphasized the need to avoid pooling different probiotic strains unless they share a common mechanism of action (2). Since the study pooled several distinct probiotics and synbiotics, the used approach directly contradicts experts' consensus recommendation to avoid such generalizations, as it can obscure specific strain effects and lead to misleading conclusions. Unfortunately, the study did not provide any justification for the pooling of different strains and types of interventions.

## 3 Inclusion errors and missed studies

Two of the studies included in the meta-analysis, specifically the study by Tayebi-Khosroshahi et al. (3) and Saxena et al. (4), did not involve an intervention with either probiotics or synbiotics, but rather with a prebiotic (lactulose syrup) and enzobiotic (synbiotics with proteolytic enzymes), respectively. This unjustified inclusion affects the validity of 6 out of 15 presented meta-analyses due to intervention misclassification.

The authors did not acknowledge or differentiate their work from previous systematic reviews on this topic, such as the Cochrane review (5) and one published in the *Frontiers in Nutrition* (6). As a consequence, they missed some studies clearly eligible for inclusion, such as three Iranian studies conducted by Abbasi et al. [testing *L. plantarum* A7 (7)], Dehghani et al. [testing synbiotic with 7 bacterial strains and fructooligosaccharides (8)], and Kooshki et al. [testing synbiotic with 1 bacterial strain and fructooligosaccharides (9)]. Thus, the meta-analyses did not incorporate all available sources of evidence.

## 4 Inconsistent application of the Cochrane Handbook guidelines

Despite claiming adherence to the “Cochrane manual” (1), the authors' choice to switch between random-effects and fixed-effect models based solely on the *I*^2^ statistic contradicts Cochrane Handbook recommendations (10). Model selection should be driven by the clinical and methodological diversity among the studies (that is definitely high due to the heterogeneity of interventions), not just the statistical measure of heterogeneity. Relying on *I*^2^ alone can lead to inappropriate model selection, affecting meta-analysis validity (10).

The main positive results of the study, reduction of blood urea nitrogen (BUN) and C-reactive protein (CRP) level were calculated and reported as standardized mean difference (SMD) instead of weighted mean difference (WMD). According to the Cochrane Handbook, SMD should be chosen “when the studies all assess the same outcome, but measure it in a variety of ways (for example, all studies measure depression but they use different psychometric scales)” (11). This choice assumes that standard deviation differences among studies result from measurement scale differences, not real population variability differences. Given that the study conducted by Chang Liu et al. (1) included populations across a wide range of CKD severity (both non-dialysis and dialysis patients), this assumption is likely violated. Moreover, the measurement methods for BUN and CRP are similar worldwide, therefore we do not see the necessity to use less intuitive SMD.

## 5 Lack of consideration of the minimal clinically important difference

The authors emphasize that certain SMDs are statistically significant, but they fail to reference the minimal clinically important difference (MCID). A statistically significant SMD does not necessarily imply that the observed changes are clinically meaningful. Without discussing the MCID, the clinical relevance of the findings remains unclear, potentially leading to overestimation of the intervention's benefits.

## 6 Protocol preregistration and adherence

Even though the authors registered the study protocol in the PROSPERO database (CRD42024526836), this occurred more than 3 months after study commencement. The protocol, written in the past tense, was submitted on March 20, 2024, while the database searches were done on December 1, 2023, and the journal submission on May 18, 2024. This timeline negates the bias-reduction purpose of protocol registration. Moreover, the authors missed to provide results for one of the primary outcomes mentioned in the protocol (hemoglobin level), whereas they provided very interesting, but not prespecified in the protocol, subgroup analyses.

## 7 Discussion

The systematic review process is critical for making informed clinical decisions. While the authors have made a commendable effort to synthesize the available data on probiotics and synbiotics in CKD, the methodological issues outlined above significantly undermine the reliability and applicability of their findings. We recommend that future analyses distinguish between different types of probiotics and synbiotics, adhere strictly to methodological guidelines, consider the clinical relevance of observed changes, and ensure that all included studies meet the eligibility criteria. Moreover, the necessity of conducting new systematic reviews should be critically evaluated, particularly when previous comprehensive reviews exist.

The study exhibits several methodological shortcomings that align with common issues in probiotic systematic reviews. These include the lack of protocol registration before study commencement, non-adhering to the published protocol, inappropriate pooling of different types of interventions, absence of a transparent list of excluded studies, and limited clinical applicability due to insufficient strain-specific analysis. These weaknesses significantly undermine the study's reliability and applicability, echoing the broader concerns highlighted in our evaluation of the systematic review landscape for probiotics (12).
